# Clinical Utility of the Canadian Occupational Performance Measure in Older Adult Rehabilitation and Nursing Homes: Perceptions among Occupational Therapists and Physiotherapists in Spain

**DOI:** 10.1155/2020/3071405

**Published:** 2020-12-10

**Authors:** Elisabet Capdevila, María Rodríguez-Bailón, Maria Kapanadze, Mariona Portell

**Affiliations:** ^1^Escola Universitària d'Infermeria i Teràpia Ocupacional de Terrassa (EUIT), Universitat Autònoma de Barcelona (UAB), C/de la Riba, 90, 08221 Terrassa, Spain; ^2^Department of Physiotherapy (Occupational Therapy), Facultad Ciencias de la Salud, Universidad de Málaga (UMA), C/Arquitecto Francisco Peñalosa, 3, 29017 Málaga, Spain; ^3^Department of Psychobiology and Methodology of Health Sciences, Universitat Autònoma de Barcelona (UAB), Campus de Bellaterra, Cerdanyola del Vallès, Spain

## Abstract

**Introduction:**

Scientific evidence that supports the psychometric properties of the COPM as a tool to enable personalized care has been repeatedly shown. However, there is a lack of studies about its utility within the Spanish research community.

**Aim:**

This qualitative study seeks to ascertain the perceptions of professionals from social health centers, nursing homes, and Spanish rehabilitation services about the clinical utility of the COPM as a standardized instrument.

**Methods:**

Thirty occupational therapists and physiotherapists in four focus groups discussed the experience of applying the COPM. The interpretative phenomenological analysis (IPA) incorporated a multidimensional model of clinical utility based on the components of acceptable, appropriate, accessible, and practicable by the clients, professionals, and institutions. *Results and Discussion*. The results of the utility study showed that the COPM helped professionals and clients to gain significant involvement in the treatment process. The COPM contributed to the process of further goal setting, occupation-based, and client-centered, thus achieving considerable satisfaction from the clients that had treatment. The professional training and adaptation to the geriatric population were vital to this process.

**Conclusion:**

The COPM is a useful and viable tool for the institutions that are supportive of a client-centered approach in the Spanish context.

## 1. Introduction

The Canadian Occupational Performance Measure (COPM) was designed to provide an occupation-focused outcome measure, and it defends the importance of the client being involved in the intervention process [1, 2]. This client-centered tool is seen as an important resource to cope with the complex problems derived from ageing and dependence situations in most countries [3].

The COPM uses a semistructured interview format directed at identifying the issues and problems of occupational performance, as well as to detect changes in a client's perception of improvement over time [4, 5]. It allows the clients to identify and prioritize issues and problems of personal care, leisure, and productivity. This instrument takes into account the context, personal circumstances, and life history of the client [6]. Besides showing prioritized problems, the initial administration of the COPM serves to guide clinical reasoning and helps to set the goals of the intervention ensuring that the clients' needs are addressed and that they consider the occupation during the rehabilitation process [4, 5, 7]. In professional practice, this measure facilitates communication between professionals and clients [1, 2].

The COPM has been translated into more than 35 languages. One of the properties that has been studied in different cultural adaptations of the COPM is its utility. We undertook the narrative literature review on this topic that included English and Spanish language studies published between 1994 and 2019. Using the databases CINAHL Complete, PubMed, ERIC, EBSCOhost, Scopus, APA PsycInfo, MEDLINE, ScienceDirect, SciELO, Dialnet, and Redalyc, we employed the following keyword searches: “Canadian Occupational Performance Measure”, “clinical utility”, “feasibility”, “rehabilitation”, “nursing homes”, and “elderly”. We found 52 articles of relevance to our study. By tuning the search strategy, the final articles were reduced to 16.

The initial studies about the clinical utility of the COPM were performed by the authors that designed the instrument [2, 8]. Lately, various researchers published studies and reviews that considered a utility perspective at the benefits for the clients as well as the improvements in professional practices, tied to different contexts and cultures [9–13]. Our review of these studies showed (a) the COPM helps the client to be aware of the existing problems and it helps to identify the goals of the intervention, encouraging the participation and adherence of the client in the rehabilitation process [1, 12, 14]; (b) it helps in developing more individualized programs, as well as helping to improve clinical decision-making and facilitating interdisciplinary work teams [1, 4, 12–17]; (c) it is useful for institutions and as a measure for the outcomes at different levels of care, and at the same time, it empowers the user for better self-management [18]; (d) as far as we know, no studies have been done on the COPM utility within the Spanish research community.

In the Spanish context of elderly care and rehabilitation, the standardized assessment instruments used in the services of occupational therapy and physiotherapy are directed at functional measurement only in terms of activities of daily living (ADL), such as the Barthel Index (BI) or the Functional Independence Measure [19–21]. Moreover, these measures usually are focused on the performance components, and it is not clear that the client needs or wants to evaluate these components [1]. The conventional approach to treatment in Spain is static and protocolized and is not producing satisfactory results, and these increase health and social care costs [22, 23]. As a response to these concerns, Spanish healthcare professionals (HCPs) and researchers propose an alternative conceptual framework that includes the active participation of clients [24, 25]. However, the switch to client-centered practice (CCP) requires an overhaul of the standardized and useful occupational therapy instruments that can be shared with the physiotherapists of the same center. Some studies underline that the shared vision towards the improvement of the functional outcomes and shared use of the COPM significantly support the quality of the interdisciplinary work [16]. The client-centered approach means professional praxis should emerge from the perspectives of patients and is core to the occupational therapy interventions [26, 27]. Moreover, the need to provide evidence on the utility of a practice centered on the client's needs has been established [5].

Commonly, clinical utility means the usefulness of intervention for, or in, clinical practice shown through clinical efficacy studies or economic evaluations [28]. The judgments over instrument usefulness should involve multiple aspects such as administration facility and time, simplicity of the format, clearness of the questions, and facility of the correction and register. Besides, it should permit an interpretation of the findings and the benefits of the use of the instrument as well as an acceptance degree of the tool by the client or therapist among other aspects [9, 14].

Possibly, Smart's (2006) multidimensional model of clinical utility created a broader vision about the concept. It conceptualizes clinical utility as a multidimensional judgment about the usefulness, benefits, and drawbacks of an intervention, encompassing four components: acceptable, appropriate, practicable, and accessible. We propose to use this model to judge the use of the COPM. The *acceptable* component includes the opinion or degree of acceptance of the assessment or intervention by healthcare professionals (HCPs), clients, and their families in the organizational context or society in general. The opinions and perceptions of all parties are essential information as well as the ethical, moral, or social implications. The *appropriate* component draws on the effectiveness of the measure in terms of relevance and adaptability to the intervention. The *practicable* component has to do with a degree of functionality and suitability of the instrument in the particular practice context. Also, it includes the need for capacity building and skill training of the healthcare personnel. The *accessible* component shows economic considerations (license purchase, training, etc.) [28].

## 2. Purpose of Study

The aim of this study was to analyze the clinical utility of the COPM in interdisciplinary rehabilitation services of Spanish social healthcare centers and nursing homes, using Smart's (2006) multidimensional model of clinical utility, specifically (1) learning about the perceptions of occupational therapists and physiotherapists about the use of the COPM as a client-centered measure, and if it was *acceptable* and *appropriate* for clients, their families, therapists, and institutions, (2) documenting the experiences of the administration and usability of the measure in everyday practice, as well as the training needs, exploring the *practicable* component, and (3) recollecting and analyzing the opinions of the occupational therapists and the physiotherapists about the *accessibility* of the measure. Besides, it was expected that the findings would solve the cost-effectiveness balance as well as the viability of the necessary resources.

## 3. Methods

### 3.1. Ethics

The present research was approved by the CST Clinical Research Ethics Committee (CREC) of the Terrassa Health Consortium (Consorci Sanitari de Terrassa) via a resolution dated July 18, 2016. It strictly followed the ethical criteria outlined, including participation consent from each center and participant.

### 3.2. Study Design

The current study follows the qualitative research tradition framed by interpretative phenomenological analysis [29, 30]. The theoretical underpinnings of IPA belong to Husserl's phenomenology and hermeneutics, combined with symbolic interactionism [29]. IPA is especially appropriate for healthcare research as it permits to relate findings to dominating theoretical perspectives and explore how clients, in our case, within focus groups, ascribe meaning to their experiences in their interaction with the practice environment. Besides, this type of analysis acknowledges that the researcher's engagement carries an interpretative element, even more if the principal investigator or research assistants are part of the focus groups [29, 31]. In this study, the phenomenon of utility was investigated in depth, based on emerging themes from data, and later on interpreted by Smart's conceptual framework [28]. The focus group technique was chosen to guide the qualitative data recollection design [31].

### 3.3. Study Sample

The participants were recruited through the professional association of Catalonia by email. The principal investigator requested professionals to express their motivation for training in the use of the COPM and research participation. After, the interested researchers undertook two informative sessions open to occupational therapists and physiotherapists of the area. A total of 30 healthcare professionals (HCPs) participated in the study: 22 occupational therapists and 8 physiotherapists, representing a total of 20 social health centers and nursing homes in Catalonia. All participant physiotherapists were working in an interdisciplinary team with occupational therapists.  Table 1 shows the participants' characteristics. It was the first time that the participants were using the COPM in their services.

### 3.4. Procedure

The procedure was divided into three phases throughout a year. In the first stage, the participants completed the training before the administration of the measure. The 8-hour training included theoretical and fieldwork sessions centered on the use and application of the COPM and the CCP. The process for the administration of this evaluation tool is available in Table 2. The facilitators explained the occupational therapy basic terminology to physiotherapists upon request. Additionally, the researchers enabled the digital platform that included the reference materials, video examples, and interactive group forum to solve the doubts and questions throughout the investigation period. In the second stage, the participants that provided a follow-up of the clients administered the COPM to more than 10 of them within the next eight months. In the third stage, the researchers invited the participants by email for the focus group discussions and grouped them by their availability. This stage included the data collection process.

### 3.5. Data Collection

The research respected the main characteristics of a focus group [31, 32]: (a) a semistructured interview sustained in a group of people about the common theme of interest proposed by the investigator, (b) the homogeneous character of the group to eliminate communication barriers, (c) moderator-expert on the topic, and (d) manageable size of the group (5–10 persons).

Previously to the sessions, the researchers developed the semistructured interview script that included general themes to respect the IPA process when guiding the focus group dynamics. All groups maintained the same structure. Each focus group session started from the presentation of general information, the goals of the sessions, and the dynamic elements (the group norms and confidentiality, the open turns, the importance of the free expression of the opinions to be able to establish the interactive dialogues within the groups, etc.). Subsequently, the facilitators encouraged the participants to reflect on three core aspects around the concept of clinical utility by (1) writing about three themes and visualizing them to facilitate thematic tracking throughout the session and (2) moderating the free opinion discussions and open interactive dialogues, emphasizing the importance of individual opinions. These three core themes or aspects were aimed to fit the specific objectives of the study: (1) the consideration of the pros and cons about the use of the instrument (acceptable and appropriate components), (2) the process of learning during the implementation (practicable component), and (3) future expectations (accessible component).  Table 3 shows the related questions that guided the focus groups.

Each session lasted approximately 90 minutes. Video recording and voice recording were done with the consent of the focus group participants. Researcher HCPs codified all materials that included personal information of the clients before the session to ensure confidentiality as well as asking for the written consents of clients. The principal investigator shared the moderation with the physiotherapist and the occupational therapy teacher trained in the COPM. All sessions were led with the support of two research assistants who observed and took the field notes of the process. The research assistants had exhaustive information about the study and the COPM and were previously trained in the focus group technique. The meeting place for two sessions was a room at the university. The other two sessions were held at the centers facilitated by the participants to fit best with the timetable and day schedule to achieve the maximum possible assistance from them. The language used was native to all participants: Catalan or Spanish.

### 3.6. Data Analysis and Quality of Data

The principal investigator ensured the literal transcription of the focus group data and the field notes using the SoundScriber program. Besides, the data were systematically sorted and uploaded using Atlas.ti 8. The key stages of IPA were respected [30]: (1) reading and rereading of the transcribed text (emerging codes and subcodes), (2) identifying and labeling themes and grouping them together as clusters (categories), and (3) introducing the structure into the analysis using Smart's conceptual framework [28].

The subsequent coding and subcoding were an iterative process guided on the basis of the contributions of authors who have published about the utility of the COPM [4, 5, 9, 14, 16] and the consensus achieved by researchers [33]. Further, these codes, subcodes, and themes were categorized based on the conceptualization proposed by Smart [28] (see Table 4). This allowed to sort the information by participants in each component.

### 3.7. Techniques to Enhance Trustworthiness

The following activities were undertaken to ensure the trustworthiness of the process: the data were verified by giving back the transcriptions to the participants of the focus groups for the revision of the content [31, 33], and the triangulation was carried out separately by a research assistant and occupational therapy researcher familiar with and trained for the use of the instrument, but objectively outside the fieldwork and the sample of the participants to contrast the citations, codes, and categories. Intercoder reliability (Atlas.ti 8) was calculated at 70% concurrence using initial coding of one sample data of the focus group that was selected at random. Using a mutually agreed upon coding scheme, our intercoder reliability was calculated at 92%. A third researcher followed the process by reading transcripts and checking appointments, codes, and categories [33]. A total of four researchers were involved in the analysis process and met regularly.

### 3.8. The Use of the COPM: Acceptable and Appropriate Components

This section addresses the findings related to the perception that the participants had about the use of the COPM as a client-centered measure, and if it was acceptable and appropriate to participants and their families, therapists, and institutions, generally.

## 4. Findings

The findings were structured following the concepts set by Smart [28]. The report attempted to reflect the authenticity of the information as much as possible, gathering textual words when both using concepts and transmitting the meaning of the message. The idea was to maintain the exact sense expressed by the participants. For each block, the differences between the workforce environments of each of them were highlighted.

### 4.1. The Use of the COPM: Acceptable and Appropriate Components

This section addresses the findings related to the perception that the participants had about the use of the COPM as a client-centered measure, and if it was acceptable and appropriate to participants and their families, therapists, and institutions, generally.

#### 4.1.1. The Participants' Opinion about the Client Experiences with the COPM

The professionals perceived that using COPM made participants feel heard. The clients and their family members expressed the gratitude for the time devoted both for evaluation and intervention:

They liked to talk a lot and be listened to. It was their time. They were grateful for this approach (P13).

Another of the highlights was that this way of assessing increased the awareness of clients about their own needs, according to participants. The way to set the scene for the interview helped to understand the life of the client in greater depth, enhancing the insight of the client about the situation.

The way of interviewing them, above all, makes them aware of the problems at the family level, harsh situations…. (P18).

Throughout the intervention, clients progressed in the level of awareness they had about their needs. Mostly, when discharge from rehabilitation was imminent, clients focused their demands on the realities of their home environment.

All participants agreed that the clients reported satisfaction with the process of goal setting which was directly impacting their motivation and making them more involved in the process. This way, the clients felt themselves being active participants in the intervention. They reported that the intervention and the results were efficient and positive. Although there was no performance improvement in many cases, the clients reported significant satisfaction in occupational performance.

The level of involvement of the patient, I think, rises when you have confronted him/her with his/her problems. When a person is prioritizing them and saying, “I want to achieve this!” the level of involvement is higher (P1).

All the participants agreed there were two essential limitations while using the COPM with the clients. The first difficulty was to identify and name the problems, needs, or aspects/goals that they preferred to work with, whether it was due to a complex emotional or cognitive state of the client. The participants attributed this to the life conditions that the person was experiencing at the time of evaluation. However, some focus group members argued that it could be due to the respect and authority granted to healthcare personnel for decision-making, especially among older people, who often accepted the loss of autonomy with resignation.

This is a generation where professionals or their children always make the decisions, and it is precisely like, “Are you asking that of me? No, you are the professional, and I am not. No, you decide” (P10).

The participants reported that the second major limitation was the difficulty in understanding and differentiating the concepts of performance and satisfaction as well as item scoring. Older adults were merely habituated to self-rate numerically and even less when grading using a Likert scale from 1 to 10. Despite facilitating the visual support of the scale, in many cases, it was essential to adapt and give qualitative examples of the meaning of each number. Some participants explained a feeling of discomfort among the clients.

I have had a vast, colossal difficulty (….) Prioritize, they did well; they knew what they wanted more, but… the occupational performance! The satisfaction item! It was difficult to explain the meaning of this item [satisfaction]! (P29).

A senior lady thought she [the therapist] was giving her an exam (...), [so the lady] decided to go out, and she told me: “Look, don't do this to me anymore”! (P23).

#### 4.1.2. The Participants' Opinion about the Acceptable and Appropriate Components of the Measure for Everyday Practice

Some of the participants recognized that the client-centered approach forms an intrinsic part of their profession, but their day-to-day practice and working years made them conform to the philosophy set in the center. At a professional level, the use of the COPM instrument supposed a significant change in the perspective and the way of working by respecting personal decisions and what had meaning and sense for a person.

That is a kind of reminder of what your work many times should consist of — at least I see it that way. It is supposed that we, as therapists, already have that internalized, but during the day-to-day practice (...) the philosophy of the institution makes you forget about these things (P23).

Although the majority of interviews required more time to administer the COPM than their previous evaluations, the participants considered that this time was crucial for having better engagement, establishing confidence, and participation of the person.

I, as a therapist, was centred more on listening to the problems and changing the assessment I always give… come on, explain what's on your mind? (P12).

It helps you to go deeper into the person, creating a space for communication necessary for acknowledging the values and beliefs of a person.

There is a change because I sit with the clients in the interviews, which many times is impossible. However, following this process, I sat with the patients, and I established another type of bonding with them. The link they had with me or with the goals was very different. Just administering the COPM was already more person-centred (P13).

The participants reported the use of the COPM helped to visualize or determine goals. It also permitted to justify the application of a particular type of treatment.

Sometimes, when you want to introduce technical aids, they are very reluctant. However, instead, if you say: “Well, since you have told me that you would like to achieve this…there is no other way…let us see if we try it with this product”. And, well, I believe there was more acceptance (P1).

Often, the participants that were observed working with the particular goals decided by the clients stimulated improvement in the intervention outcome. They reflected the reason could be that the person does with what he or she wants or desires, not what was imposed by others. Moreover, relying on their self-report, the COPM permitted to visualize the changes, especially related to rehabilitation that allowed obtaining more specific evidence about the therapeutic work.

The other day, I checked the data collection register, and I was happy seeing the results of the intervention. I could observe the Barthel had the threshold effect so high, and the COPM showed specific changes in the occupational performance. I believe that for the research (...) it presents a scope of interest for therapists (P16).

#### 4.1.3. Acceptable and Appropriate Components of the COPM for the Institutional Context

The participants reported most centers continued working from a mechanistic paradigm in a hierarchical and authoritarian way, where the institution gave the limits, and the services set the program objectives and goals for the intervention. The participants argued the executive directors of the center should commit themselves to promote the change of paradigm and the structural reforms. In general, they considered that including the COPM as an interdisciplinary evaluation tool would have permitted to recollect the personalized data and raise the realistic goals of the interventions. The participants discussed this would have supported the establishment of the CCP in the center and would have generated evidence and improvements in treatment.

They must be convinced; they must be and there should be a commitment to an idea with the changes that this implies (P5).

It must follow the philosophy of the center (…) and professionals (…) if they do not understand that you are there for the goals of the patient and not for those of the institution … You are bumping into the wall (P16).

The participants with more years of experience at the same institution had difficulty visualizing how the client-centered approach could be implemented. Notably, it was evident within the rehabilitation services, where the biomedical model was implemented for many years. Furthermore, at a social level, the participants discussed people were already accustomed to the offer of specific services, and they tended to adapt to the possibilities they might have offered.

There must be a change at the social level, at the overall level (…) This treatment that we want to offer at the level of the nursing home, the CCP, I see it as more precise or more applicable, but at the hospital level … [thinks] … resources, you have particular hours, you have a specific workload. I do not know (P12).

One of the critical aspects the participants contemplated for being able to use the COPM was the degree of implementation of the CCP in the center. Even though all reported having some training about the client-centered practice encouraged by the institution, only several participants noted the services and centers adopted this approach in reality.

The CCP is a fundamental right… it is supposed (P4) …the theory, yes, but in practice (P1).

The CCP is widely discussed…but, in reality, it could not be easily incorporated into practice (P1).

Few professionals commented on the contribution of the specific means and actions of support by the institution to implement the CCP. Currently, it is not possible to work out many objectives due to either institutional norms or the lack of resources.

### 4.2. The COPM as a New Tool: Practicable Component

This section addresses the findings related to the opinion of the participants about administration details of the measure, everyday use, and training needs of the occupational therapists and physiotherapists.

In most cases, the COPM was administered in one session. The participants reported the administration of the COPM in two sessions in cases where the client had difficulties, as they were tired physically or emotionally.

Due to the difficulty mentioned earlier in understanding and evaluating the concepts used in the tool, many therapists opted to adapt the COPM to the characteristics of the client, change the concepts to words of daily use, and, in the case of the rating, transform the numeric into qualitative evaluation. In some cases, they used visual icons (faces).

Initially, the professionals reported the time dedicated to passing the COPM was around 1.5 hours. The time oscillated, more or less, depending on the client's particular situation and needs: whether the administration was taking place in a nursing home (30-60 min in cases of elderly with complex needs) or a rehabilitation center (15-20 min). The time decreased when the therapists mastered the practice. The use of the measure implied the process of training and learning. The participants confirmed that they gained the following skills: the ability, confidence, and naturalness along with the regular application of the measure. The main aspects of these skills, besides instructional and methodological parts, were the application of ethics and practice-based evidence. The participants illustrated these observations through various examples of key skills: (1) determining the appropriate time for the administration, (2) knowing with whom it can be applied, (3) guiding the interview when the person is not aware of the problems, (4) confronting the reality of clients without violating them, (5) adapting the measure to the characteristics of each person, (6) splitting out and shaping the goals, explained by the client, and (7) knowing how to graduate the intervention or to regulate the degree of involvement of the family.

The first interview I did was a difficult experience, because there was a lot of effort, a lot of time, and, above of all, I wasn't confident with the tool (P13).

All groups commented that it was essential that the client was minimally adapted to the environment of the center at the time of the first assessment. In the case of rehabilitation, the average number of days for the adaptation was two or three, and in the nursing homes, this number was extended to the first weeks. In some social healthcare centers, the therapists proposed using the COPM in the middle of the overall treatment process. The underpinning argumentation of this new vision was the orientation of the goals to the discharge.

We are holding the meeting with the family members in the middle of the intervention, at approximately two months (P16).

The participants underlined that they gained skills through the training offered by the principal investigator and that having available the digital platform with the materials as well as further follow-up of the questions that might have raised in the practice context was helpful. Based on the self-report, the participants improved in the parameters of ability, security, and fluency when administrating the COPM.

At the end of the study, the participants expressed many doubts. These questions conveyed a general interest in proper application of the COPM, and some requested more training in certain aspects of the theoretical foundations and practical abilities.

Regarding future use of the COPM, most participants expressed their motivation to use the measure partially or entirely, depending on the specific cases. They believed the attitudinal change in the institutional context and the interdisciplinary team could have promoted the incorporation of the measure into day-to-day practice. Notably, all of them expressed the intention of adopting implicit elements of the COPM, such as listening, asking about the client's desires and priorities of their occupational performance, or clarifying and recalling the therapeutic goals.

It means acting more for what one wants … let us not impose our goals … if not, keep in mind, above all, what each person really wants (P15).

### 4.3. The Cost-Effectiveness and Viability of the Necessary Resources: Accessibility Component of the COPM

This section reflected the findings related to the opinion of the participants about the cost-effectiveness and viability of resources that are necessary for the successful application of the COPM in the practice context.

Although the institutions can easily acquire the COPM, most of the participants highlighted key resource constraints for making the use of the measure broadly accessible. These were the low personnel ratio, the short time of admission of the patient in the case of rehabilitation centers, and poor infrastructure of the institution.

Of course, I find that in day-to-day practice, you go in such a hectic manner that during the first evaluation I am already intervening [Everybody: “Yes, yes, yes”.) (P3).

Before the time for convalescence was two months, and now they are pushing you to do it in four weeks (P12).

The time is one thing, and the resources are another (…) For example, there is no kitchen. These are things that are very meaningful for them and very basic; it is their day-to-day life that they want to keep going (P25).

Or if you need to provide supervision and there is no staff, and you cannot do it (P24).

Sometimes 10 minutes or a quarter of an hour more here, you will do a lot of work for a while, no? [“Yes, yes, of course” (P14)]. But sometimes you will not (P10).

The participants reported that the acquisition of the COPM and the improvement in terms of necessary resources as infrastructure, available time and human resources, would help the quality and efficiency of the intervention as well as professional satisfaction in the long term.

## 5. Discussion

This study is aimed at exploring the clinical utility of the COPM in interdisciplinary rehabilitation services of Spanish social healthcare centers and nursing homes, mainly focusing on the perceptions of the occupational therapists and physiotherapists. This section discusses the main findings by grouping them using the multidimensional model of clinical utility of Smart [28].

Explorations about *acceptable and appropriate* components show the major findings indicated that the use of the COPM implied an attitudinal change of the interdisciplinary teams towards a more holistic and client-centered approach. The participants reported these views helped the teams to recover humanistic principles, respect, collaboration, and recognition of self-determination. They recognized the client as an active agent in the process and acknowledged respect for autonomy as a fundamental value [26, 27].

One of the aspects most valued by the clients and their families was the time for listening and the dedication by the healthcare personnel, which is seen as an example of quality healthcare. The semistructured interviews generated a depth that helped the clients to improve their degree of awareness about their situation [16] and thus identify their problems [5, 10, 34]. There were doubts among the participants about the appropriate timing for the interview. The initial study of utility by Toomey indicated the difficulty of gaining insight in some acute situations as well as cognitive or emotional states while using the measure [17]. The same author suggested choosing the best time to administer the tool, always under the criteria of the professional [2]. Therapists should keep in mind that the clients need to have minimal capacity to establish a conversation with the professionals [5].

The difficulties in identifying or naming problems [2] in many cases were the themes linked to the lack of habit. Traditionally, the professional defined the problem and recommended types of interventions. For some clients, a client-centered perspective implied a change that was difficult to assimilate, or they could perceive it as a lack of professional competence. They expected a professional to be an expert [12]. The nondirective approach, especially in older adults, is still perceived as less valuable due to past experiences as passive recipients of services. In addition, some clients felt confused and insecure because of the many questions from the professional [1]. Moreover, the revision of Stevens et al. showed that older adults were reluctant to express their concerns because of their fear that professionals could interpret the low-scored items as a complaint [12].

The participants agreed that giving primacy to the clients and empowering them to create the bonds of mutual trust allowed to obtain the relevant information and demands based on their desires and needs. These were always real and genuine/authentic, as considered by Bodiam [35], and could not be obtained in other types of assessments. The open questions about clients' needs permitted to contemplate new dimensions and a broader spectrum of aspects of the occupational life of people [5]. The in-depth character of the interview helped to discover meaning and sense for the client, and the process itself became meaningful. This fact reflected the idea that the clients had exceptional knowledge of their occupational lives. They know their needs better than anyone [26]. The CCP offered and enhanced the opportunities for them to participate in their occupational lives, opposed to feeling deprived of performing meaningfully, so the goal and the intervention got oriented to the occupation [4, 5, 15]. The instrument was appropriate for the detection of changes in occupational performance [10].

The participants reported having personal paternalistic views and beliefs that served as a limitation to client-centered practice and when defending the client's personal convictions. Similar to the findings of Colquhoun et al., they assumed they knew what would be the best for the clients or what they would be able to do. Parker and Sykes considered this dynamic [10, 36]. Previous studies showed that it had to do with the adoption of the new paradigm, the difficulty, and the dedication time needed to accept this new perspective, even if the professionals had previous training [1, 2]. This highlighted the importance of obtaining theoretical knowledge of the Canadian Model of Occupational Performance and Engagement (CMOP-E) to understand the theoretical foundations of the instrument [26, 27]. From this point of view, we recommend the involvement of professionals in continuous education.

Concerning the *practicable* component, as also observed in the present study, Parker and Sykes indicated that applying the COPM with clients helped to develop competencies (enabling/empowerment) [10]. These skills permitted them to participate actively in the selection of and decision-making about their goals and bring clear motivation and involvement in the treatment process. As highlighted in previous studies, this could have created a unique engagement with active participation in the intervention of more relevant occupational issues [14]. Additionally, it would have allowed more efficient results [34].

The considerations mentioned above emphasize the aspects that go beyond the mere administration of a measure. Knowledge of the interviewing techniques, competencies of communication, and clinical reasoning are needed to reach consensus with the client to generate coherent and realistic therapeutic goals. Only experience, training, and daily praxis can help the professional to acquire these competencies and feel comfortable with the instrument. This shows the necessity for each therapist to find personal strategies when conducting the interview [1, 2, 5, 14]. Moreover, like any evaluation instrument, it requires learning and practice [36].

Regarding the *accessible* component, it was crucial to keep in mind that many participants reported that in specific contexts and with particular cultures, the integration of the CCP happened at different levels [37]. However, the participants confirmed that institutions with hierarchical and protocolized structures offered few opportunities to carry out meaningful activities. This inevitably led to the institutionalization of the clients. The clients got habituated and never demanded or protested. Added to the specific characteristics of older adults, many with complex needs from disabling pathologies or low resources, the accommodation to this situation many times deprived them of meaningful performance and, further on, alienated them from significant life experiences. The findings showed the use of the measure promoted and reinforced the CCP [10, 14]. Besides the clients, their families, and the interdisciplinary teams accepted the measure, its usefulness in everyday practice should imply the process of cultural adaptation at all levels (personal, professional, social, attitudinal, and institutional).

The routine application of the COPM in the future is complex, although all professionals who participated in the study were constant in its use [1, 4]. Some factors that go beyond the decisions and opinions of the therapists condition the future use of the COPM. However, all professionals expressed convictions about its efficacy. According to Toomey, the therapist needs to have the support of the institution/management teams to integrate the CCP and to promote efficient use of the COPM [17]. It is evident that the needs and motivations of the therapists are fundamental factors for bringing changes to their practice. However, researchers agree that these decisions will always be impacted by work contexts [28]. Working on goals prioritized by the clients made the institution acquire more materials and resources, improving spaces to complete different tasks (for example, accommodate the areas to make garden activities). Administration of the COPM required additional time [5, 17]. However, Wressle et al. established that [18] “a focus on problems that are important for patients makes more efficient use of rehabilitation resources”.

### 5.1. Future Lines of Research

The current study derived in several future lines of research. Some were formulated by the therapists who participated in the study, such as an adaptation of the COPM to the older adult population with mild cognitive difficulties and/or few personal abilities, which was also underlined in other studies [1, 4, 5, 12, 13, 16, 18]. This kind of adaptation was discussed by other authors, such as Toomey [17], and could be extrapolated to the different fields of pediatric care or mental health, where the characteristics of the clients can present similar difficulties.

In general, all participants expressed satisfaction with their role and being able to work with the goals desired by the client. This underlines a mutual satisfaction with the process. It would be interesting to analyze the relationship between the COPM implementation and the improvement of work engagement, which is one of the main elements in the prevention of burnout.

This study revised the components related to the clinical utility of the COPM. The researchers will examine other psychometric properties of the instrument like validity, reliability, and responsiveness in subsequent studies with Spanish samples.

It will be a challenge to explore the multidimensional clinical utility, integrating the voices of other key actors implied in the change [28, 36]. It would be preferable to contrast and triangulate the opinions of the family members and caregivers or receive a contribution from the institutional perspective. Also, it could be interesting to use a framework of indirect observation with diachronic analysis in order to detect how the interaction of the key actors changes in response to a greater COPM use [38, 39].

The authors propose to continue working on, exploring, and resolving the detected limitations related to the institutional and healthcare policies that are usually alienated from the day-to-day professional reality and praxis.

### 5.2. Limitations

This study focused on the self-report that professionals gave on how the clients have perceived the COPM. The authors could not get direct self-reporting from the clients but tried to solve this limitation, ensuring that they engaged the clients and encouraged them to share their opinion.

Another limitation of the study was particular to the qualitative tradition: the researchers could avoid posing the direct question about the difficulties or problems related to the use of the measure, focusing only on the generic themes. This decision was made to obtain concrete answers and to respect the time inversion of the clients, but it was clear that each question can condition the response. We tried to ensure the trustworthiness of the study by other mechanisms described in the article.

All the participants were occupational therapists and physiotherapists interested in the COPM; therefore, the study was not pretending to generalize the results to other professionals. It contemplated the diversity of the centers and interdisciplinarity, but the integration of other professional profiles or professionals of the centers who had previous interest would have enriched the research. There was an attempt to compensate for the biases provoked by the pressure of the group dynamics or by the researcher following the principle of reflexibility. Researchers ensured that the IPA was completed correctly. The centers were combined so that the participants did not know each other, and some of them did not know the principal investigator [33].

## 6. Conclusion

This study analyzes the complexity of factors involved when introducing a new measure into Spanish health services. Numerous studies from other countries established that the main limitations in the use of the COPM involved the clients and the experience of the professional [23]. However, the current investigation pointed out that the principal limitation had to do with institutional policies and services. The results showed multiple benefits for everyday practice and the aspects that motivate clients, professionals, and institutions when applying the COPM in the overarching framework of the client-centered practice. One of the significant challenges is to advance and opt for what the professionals and clients believe in, see, and consider as beneficial, thus supporting social progress in general.

## Figures and Tables

**Table 1:** Participant characteristics (*N* = 30).

Variable	*M* (SD) or *n*	Range
Age in years, *M* (SD)	35.5 (5.4)	27–44
Gender		
Female	28	
Male	2	
Years working as an occupational therapist/physiotherapist	14 (6.4)	4–25
Work setting		
Older adult rehabilitation	15	
Home care	15	
Type of center		
Private or semiprivate	22	
Public	8	
Professional		
Occupational therapist	22	
Physiotherapist	8	

**Table 2:** Process for the administration of Canadian Occupational Performance Measure.

Step 1	Problem definition	The initial semistructured interview that addresses daily routines when identifying what the person wants, needs, or has to do in the three areas of occupational performance proposed by the Canadian Model of Occupational Performance^26,27^: self-care, productivity, and leisure.
Step 2	Rating importance	Ask the person to rate on a scale from 0 to 10 how important each of these problems is.
Steps 3 and 4	Scoring	Choose the five most prioritized issues in terms of the importance given by the person.
		Ask the person to rate on a scale from 0 to 10 their performance of each activity (degree of performance) and their satisfaction with the way they are carried out (degree of satisfaction).
Step 5	Reassessment	After carrying out the intervention/rehabilitation with the client, administer the performance and satisfaction scales again.

**Table 3:** Clinical utility conceptualization, themes, and focus group questions.

Conceptualization proposed by Smart (2006)		Focus group questions
Component	Aspects	
Acceptable	To professionals	(1) Use of COPM (a) What advantages/positive aspects/benefits have you had as a professional when using the COPM during the intervention? What advantages were associated with the client/family relationship, or with the interdisciplinary team of the center/institution? Does it work as expected? Was there any impact on the intervention? (b) What difficulties/problems did you find (or do you see in the present) as a professional when using the COPM during the intervention? What difficulties were associated with the client/family relationship, or with the interdisciplinary team of the center/institution?
	To clients (including families and/or caregivers)	
	To society (public or stakeholder groups)	
Appropriate	Effective	
	Relevant	

Practicable	Functional	(2) Learning process (c) How would you describe learning during the process of administration of the COPM? What elements favored or hampered this learning?
	Suitable	
	Training or knowledge	

Accessible	Resource implications	(3) Future expectations (d) Could you describe the future use of the COPM? Do you intend to incorporate the measure into your day-to-day practice? Why would you do so? Could you propose the aspects of the improvement?
	Procurement	

**Table 4:** The interpretative phenomenological analysis of the obtained data.

Focus group interview themes	Codes	Subcategories	Categories	Component groups
Use of COPM	Awareness (benefits) (27)	Benefits or limitations expressed by the client or the family: client characteristics (age; physical, cognitive or emotional states; social and cognitive aspects); comprehension of the role of OT; participation of the client; the difficulty of grading; awareness; need and problem identification, and goal setting; and implication in the treatment process.	Advantages: clients and family	Acceptable and appropriate
	Listening (benefits) (8)			
	The implication/participation in the intervention process (17)		Disadvantages: clients and family	
	Result improvement (10)			
	Characteristics of the client (13)			
	Unawareness of the professional role (3)			
	Emotional state (3)			
	Cognitive deterioration (3)			
	Difficulty in scoring (28)			
	Identification of the problems and goals (15)			
	Institutionalization, conformism (8)			
	Client-centered practice (15)	Benefits or limitations (a) perceived by the professionals: the effectiveness and efficacy of the intervention; occupational performance problem identification; setting the goals; efficiency of the interview; evaluation of the results of the intervention; register of the changes; and decision-making process during the intervention; (b) related to knowledge and skills in CCP; and (c) related to the characteristics of the workplace (contract types, resources available, team, and the role of OT).	Advantages: OT, PT, and the interdisciplinary team	
	Active listening (6)			
	Major dedication to the case/attention quality improvement (20)			
	Professional role and the team (8)		Disadvantages: OT, PT, and the interdisciplinary team	
	Knowledge about the client, his/her needs and goals (16)			
	Competences related to use of the measure (15)			
	Intervention effectiveness/results (6)			
	Personal satisfaction (4)			
	Limitations CCP (11)			
	Environment/institution (6)			
	Resources (8)			
	Availability of time (11)			
	Social health/residencies characteristics (2)	Benefits or perceived limitations related to their institution: philosophy or culture of the center; the grade of the implementation of the CCP; cultural and social levels; job and work organization; general cultural and social levels; resources; and evidence.	Advantages: institution and society	
	Grade of implementation of the CCP (18)			
			Disadvantages: institution and society	
	Generation of the change/evidence (3)			
	Context (6)			
	Resources (14)			
	Social/cultural implications (3)			

Learning process	Characteristics (4)	Characteristics of the process of administration of the measure: How? (during the session, two different sessions, individual patient particularities/reactions, need in adapting the grading process, etc.) Where? (department, room, with or without family, etc.) When? (first time, first week, etc.).	Administration process	Practicable
	Sessions/administration (3)			
	Need in explication/adaptation (7)			
	The time of administration (9)			
	The moment of administration (7)			
	The process of learning (49)	How was the learning process perceived? (changes at the initial stage, initial difficulties, etc.) Reliability.	Learning process	
		The utility of the information, of the support provided, and of the materials and videos.	Training	
	Interdisciplinary teamwork (26)	The implication of the use of the measure to the teamwork (changes, level of the interest, participation, and renegotiations of the professional limits).	Teamwork process	

Future expectations	Future (55)	Use of the measure starting from the research closure. Application in the center or departments of OT; interest of the center.	Future	Accessible
	The occasionality of incomes (23)	Includes resources needed for the measure to be used: economic and infrastructure/time/personal/materials.	Resources	
	Professionals resources (7)			
	Infrastructure (7)			

## Data Availability

Access to data is restricted. We are according to the Spanish data protection legislation not allowed to submit the data or give access to the data used for the analyses.
